# Screening Active Compounds from *Garcinia* Species Native to China Reveals Novel Compounds Targeting the STAT/JAK Signaling Pathway

**DOI:** 10.1155/2015/910453

**Published:** 2015-05-18

**Authors:** Linfeng Xu, Yuanzhi Lao, Yanhui Zhao, Jian Qin, Wenwei Fu, Yingjia Zhang, Hongxi Xu

**Affiliations:** ^1^School of Pharmacy, Shanghai University of Traditional Chinese Medicine, Shanghai 201203, China; ^2^Engineering Research Center of Shanghai Colleges for TCM New Drug Discovery, Shanghai 201203, China; ^3^Cell Biology Group of Shanghai Chempartner Ltd, Shanghai 201203, China

## Abstract

Natural compounds from medicinal plants are important resources for drug development. In a panel of human tumor cells, we screened a library of the natural products from *Garcinia* species which have anticancer potential to identify new potential therapeutic leads and discovered that caged xanthones were highly effective at suppressing multiple cancer cell lines. Their anticancer activities mainly depended on apoptosis pathways. For compounds in sensitive cancer line, their mechanisms of mode of action were evaluated. 33-Hydroxyepigambogic acid and 35-hydroxyepigambogic acid exhibited about 1 *μ*M IC50 values against JAK2/JAK3 kinases and less than 1 *μ*M IC50 values against NCI-H1650 cell which autocrined IL-6. Thus these two compounds provided a new antitumor molecular scaffold. Our report describes 33-hydroxyepigambogic acid and 35-hydroxyepigambogic acid that inhibited NCI-H1650 cell growth by suppressing constitutive STAT3 activation via direct inhibition of JAK kinase activity.

## 1. Introduction

Compounds from natural herbs are important sources of drugs against a wide variety of diseases, including cancer [1]. For the last several decades, natural products have played a very important role as chemotherapeutic agents, either in their natural forms or in synthetically modified forms [2]. For instance, many agents with plant origins (paclitaxel, vinblastine, vincristine, camptothecin, and others) have already been applied as anticancer therapies. Natural compounds are considered to have a large degree of “drug likeness” because it has been suggested that most of them have some receptor-binding capability [3]. In addition, traditional Chinese medicine (TCM) provides an enormous variety of medicinal plants based on thousands of years of experience. With the understanding of the molecular mechanisms of cancer therapies and carcinogenesis, the development of chemotherapeutic agents from natural products is currently being accelerated by collaborations between medicinal chemists and biologists [4, 5].

In recent years, researchers obtained more than 120 caged xanthones from plantsof the* Garcinia* species, and most of them exhibited various potentially useful biological activities, such as anticancer, anti-HIV-1, antibacterial, anti-inflammatory, and neurotrophic activities [6]. Our group has focused on identifying bioactive compounds from* Garcinia* plants for a decade. We have collected all of the* Garcinia* plants in mainland China and used bioactivity-guided fractionation to obtain many active compounds [7]. We found that* Garcinia* species contained many special compounds, including xanthones, benzophenones, bioflavonoids, and biphenyls. By using different bioassay platforms, we were able to screen novel compounds targeting various signaling pathways. For example, a cell proliferation assay (such as SYBR green assay) identified some cytotoxic polyprenylated xanthones from the resin of* Garcinia hanburyi* [8]. By expressing a biosensor for caspase-3 cleavage in HeLa cells, we screened for compounds targeting apoptosis [9–11]. Recently, we found that oblongifolin C was an autophagic flux inhibitor using a GFP-LC3 expression screening platform [12]. In addition, we also found that the* Garcinia* species contained active compounds with antienteroviral activity [13]. Our previous studies suggested that the compound “libraries” from* Garcinia* plants comprised many active components with a variety of effects on cellular function. To better understand the antiproliferation activities of these compounds, it is necessary to perform a cell viability assay using multiple cell lines to establish their cytotoxicity. A screen using multiple cancer cell lines will provide essential information to elucidate the possible mechanisms of action of the active compounds.

In this study, we comprehensively analyzed the cytotoxicity of* Garcinia* compounds that we have obtained in previous studies. Among these compounds, five caged* Garcinia* xanthones were found to exhibit the strongest antiproliferation activity against NCI-H1650 cell. In addition, NCI-H1650 cell contained high endogenous JAKs activity. We then investigated their mechanisms of action on NCI-H1650 cell cycle arrest and apoptosis. Our results provided evidence that two of them (33-hydroxyepigambogic acid and 35-hydroxyepigambogic acid) were perhaps specific JAK2 and JAK3 inhibitors. Our data provide profound information on the anticancer activity of the main components from* Garcinia* plants.

## 2. Materials and Methods

### 2.1. Cell Panel Screen

All cells were obtained from the cell bank of the American Type Culture Collection (ATCC) and cultured in the supplier's recommended media supplemented with 10% FBS. The cell panel screen was measured using the CellTiter-Glo assay (Promega, Madison, WI) following a standard protocol. Briefly, cancer cells were seeded in 96-well plates and cultured overnight at 37°C with or without 5% CO_2_ in an incubator. Each compound was dissolved with limited DMSO and diluted to a certain concentration with culture medium and then added to the corresponding well of the cell plate. The final concentration of compound was 20 *μ*M. The control cells (without treatment) received the same amount of DMSO and were incubated under the same conditions. After 72 h cultivation, 100 *μ*L of the CellTiter-Glo reagent was added directly to each well for 10-minute incubation. The plate was then read on a FlexStation 3 microplate luminometer (Molecular Devices, USA) to monitor the luminescence signal generated by the luciferase-catalyzed reaction of luciferin and ATP. The percent of growth inhibition was calculated using the following formula: (untreated cells − treated cells)/(untreated cells − background without cells) ∗ 100.

### 2.2. Cell Proliferation and Cell Viability Assay

For proliferation assays, the CellTiter-Glo assay (Promega, Madison, WI) was used to evaluate the proliferation of cancer cells treated with test compounds as described above. Each compound was dissolved in limited DMSO and diluted to various concentrations with culture medium and then added to the corresponding wells. A dose-response curve was plotted, and the IC50 was calculated using XLfit software.

For the cell viability assay, the cells were examined by counting (in duplicate) cells that excluded trypan blue dye. The cells were seeded in 6-well plates and cultured overnight at 37°C with 5% CO_2_ in an incubator and treated with different concentrations of the compounds. At 0, 24, 48, and 72 hours, the live cells were counted using a Vi-Cell counter (Beckman).

### 2.3. Soft-Agar Colony Formation Assay

NCI-H1650 cells were plated in soft agar (Takara, Japan) at a density of 4000 cells/well in a 96-well plate. For the base layer, 1.2% agar stock solution was melted in an autoclave, cooled to 40°C in a water bath, and then mixed with RPMI1640 medium to obtain a solution of 0.6% agar in RPMI1640 containing 10% FBS. A cell suspension placed on the top layer was composed of RPMI1640 with 10% FBS and 0.4% agarose in each well. Then, the cells were incubated overnight. Compounds were added into the wells, and the NCI-H1650 cells were incubated for one week. After 7 days, alamar blue was added to the agar plate wells containing NCI-H1650 cell, and the plates were returned to the incubator. The fluorescence intensity (excitation 560 nm, emission 590 nm) was measured after 24 h.

### 2.4. Caspase-3/7 Activity Measurement

Caspase-3 and caspase-7 activities were examined using the Caspase-Glo 3/7 kits according to the manufacturer's protocol (Promega, Madison, WI). Briefly, cells were plated and grown overnight in 96-well plates (8 × 10^3^ cells/well) and untreated or treated with compound for 8 or 24 h. The cells were then incubated with 100 *μ*L of Caspase-Glo 3/7 reagent per well at room temperature for two hours. The caspase activity was determined by caspase substrate luminescence and recorded using a FlexStation 3 microplate luminometer (Molecular Devices, USA).

### 2.5. Cell Cycle Distribution Analysis

The cell cycle distribution was analyzed by flow cytometry (BD FACSCalibur, USA) with DNA staining. After treatment for 24 hours, cells were washed with phosphate buffered saline (PBS), trypsinized, and washed twice with ice-cold PBS and centrifugation at 800 g for 5 min. After overnight incubation with 70% ethanol at −20°C, the cells were washed twice with PBS. The cell pellets were resuspended gently in PBS containing 50 *μ*g/mL of propidium iodide (PI) and 10 *μ*g/mL of RNase A. The cells were incubated for 30 min in the dark and then the stained cells were analyzed using ModFit software.

### 2.6. Western Blot Analysis

Floating and adherent cells were harvested and treated with ice-cold RIPA lysis buffer (50 mM Tris-HCl, pH 7.5, 0.5% cholic acid, 2 mM EDTA, 10% glycerol, 150 mM NaCl, 0.1% SDS, and 1% Triton X-100) containing complete Mini Protease Inhibitor Cocktail (Roche Applied Science, Indianapolis, IN). Proteins were separated by sodium dodecyl sulfate polyacrylamide gel electrophoresis and transferred to nitrocellulose membranes. Membranes were blocked with 5% BSA in washing buffer (100 mM NaCl, 10 mM Tris-HCl, pH 7.5, and 0.1% Tween-20) for 1 hr at room temperature and incubated with the respective primary antibodies in 5% BSA in washing buffer overnight at 4°C with shaking. The membranes were probed with the following antibodies: p44/42 (Cat#9107, CST), phospho-p44/42 (Cat#4370, CST), AKT (Cat#9272, CST), phospho-AKT (ser473) (Cat#9271, CST), STAT3 (Cat#9139, CST), and phospho-STAT3 (Tyr705) (Cat#9131, CST). Membranes were then washed and incubated with anti-mouse or anti-rabbit fluorescent secondary antibodies (LICOR, US) diluted in washing buffer for 1 h at room temperature with shaking. Protein bands were detected using an Odyssey Imaging system (LICOR, US).

### 2.7. RNA Isolation and Quantitative Real-Time PCR

The primers for PUMA (Cat. Hs00248075_m1), CDIP1 (Cat. Hs00924663_g1), and NOXA (Cat. Hs00560402_m1) for quantitative PCR were all purchased from Invitrogen. Dissociation curves and no-cDNA controls were generated for each primer pair to detect nonspecific amplification. A standard curve was generated for each primer pair as well as for glyceraldehyde-3-phosphate dehydrogenase (GAPDH; using in this case a predeveloped TaqMan assay), to which gene expression levels were normalized by a comparative threshold cycle method. Finally, a ratio was calculated comparing normalized gene expression values in treated versus untreated controls for each sample.

Total RNA was extracted from the NCI-H1650 cell line using the RNeasy kit (Qiagen). cDNA synthesis was performed using Superscript II RNase H– reverse transcriptase (Life Technologies, Bethesda, MD, USA) to transcribe 2 *μ*g of total RNA primed with 1 *μ*L of 500 *μ*g/mL random hexamers. For quantitative real-time PCR analysis, an ABI TaqMan assay (HS00378697) was used in an ABI 7300 system with the following profile: 95°C for 10 min, followed by 40 cycles of 15 s at 95°C and 60 s at 60°C. PUMA, NOXA, and CDIP1 mRNA levels were normalized by comparison to GAPDH RNA levels in the same samples. Each measurement was performed at least in duplicate.

### 2.8. Apoptosis Induction Assay

Apoptosis induction assays were performed after 8 or 24 h incubation with compounds using the Cell Death Detection ELISA assay (Roche). Apoptosis induction is represented relative to DMSO-treated controls (set at 1.0). Error bars denote the SEM.

### 2.9. JAK Family Kinases Activity Measurement by Microfluidic Mobility Shift Enzyme Assay

The microfluidic mobility shift enzyme assay was performed in an on-chip incubation mode on a Caliper LabChip 2000 Drug Discovery System (Caliper Life Sciences, Hopkinton, MA). The extent of inhibition by the compounds is measured directly by quantifying the level of both unphosphorylated peptide substrate and phosphorylated product after electrophoretic separation using Caliper's LabChip assay technology. To evaluate the compounds for their target and the mechanism by which the compound affects JAK enzyme activity, the compounds were tested against JAK family kinases (JAK1, JAK2, JAK3, and Tyk2) with the Caliper microfluidic mobility-shift platform. The JAK family kinase enzymes and the FAM-labeled substrates were purchased from Carna Bioscience, Inc. (Japan) and Caliper Bioscience (US), respectively.

For JAK kinase activity measurement, the test compounds were diluted freshly with assay buffer (100 mM HEP ES, 10 mM MgCl_2_, 1.5 mM betaine, 0.015% Brij, and 25 mM *β*-glycerophosphate) and added to 384-well plates. FAM-labeled peptide substrate and ATP in assay buffer were added to the wells. After that, JAK enzymes prepared in assay buffer were also added to the wells and mixed to start the reaction. After a period of incubation at room temperature, the reaction was stopped by stop solution (500 mM EDTA). Finally, the plate was put on a Caliper LabChip 2000 system and a droplet of the reaction mixture was applied for electrophoretic separation in the chips of the machine. The enzyme conversion data then was read out for analysis.

### 2.10. Compounds Library and Materials

All of the compounds were obtained from our laboratory and originated and isolated from the different plant parts of 8 species of the genus* Garcinia* (Guttiferae) collected from the south of China (see Table S1 in Supplementary Material available online at http://dx.doi.org/10.1155/2015/910453). All chemicals and consumables for experiments were purchased from Sigma-Aldrich. All of the media for cell culture were purchased from Invitrogen Life Sciences. The antibodies were ordered from Cell Signaling Technology. The enzymes and the FAM-labeled substrates were obtained from Carna Bioscience, Inc. (Japan).

## 3. Results and Discussion

### 3.1. Antiproliferation Properties of Natural Compounds Library from* Garcinia* Species

In current drug market, more than 50% of the drugs discovered within the past 25 years were directly or indirectly from natural products [14]. Thus, there is growing interest in the possible therapeutic potential of natural products against a variety of ailments. Because natural compounds are considered to be affordable and safe, many potential compounds are now in different phases of clinical trials. In the past decade, we focused on applying different screening platforms to search for novel anticancer compounds from* Garcinia* plants. The natural compound library mainly includes polycyclic polyprenylated acylphloroglucinols (PPAPs), benzophenones, xanthones, and caged xanthones [15]. In this study, we profiled all of the natural compounds that we isolated from* Garcinia* plants and placed in our compound library using cell viability-based screening.

**Figure 1:**
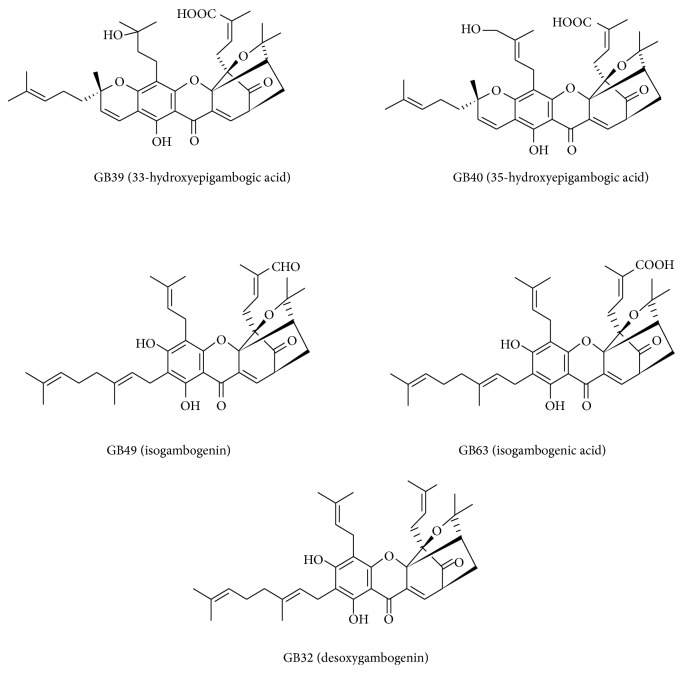
The chemical structure of the five chosen compounds (GB32, GB39, GB40, GB49, and GB63).

**Figure 2:**
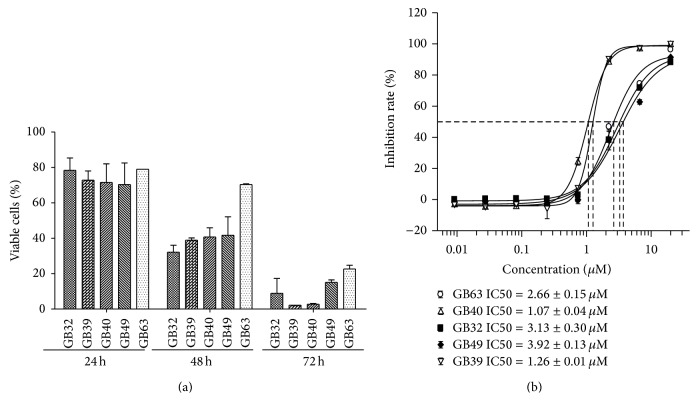
The inhibition effect of the five chosen compounds (GB32, GB39, GB40, GB49, and GB63) on NCI-H1650 cells. (a) Inhibitory effects of compounds on the viability of NCI-H1650 cells assayed by trypan blue exclusion. Cells were treated for 24, 48, and 72 hr, and the viable cells were identified by trypan blue exclusion and counted. The IC50 values were calculated using XLfit software and a four-parameter model. NCI-H1650 cells were treated for 72 hr with compounds at concentrations ranging from 20 *μ*M to 0.009 *μ*M in serial 3-fold dilutions. (b) Cell colony formation curve after compound treatment measured using the Alamar Blue reagent from Invitrogen. The IC50 values were calculated using XLfit software and a four-parameter model.

The cytotoxicity of 64* Garcinia* compounds (Table S1) from our previous studies was evaluated in panel of 35 cancer cell lines, which included lung, breast, urinary bladder, uterus, brain, liver, pancreas, stomach, colon, kidney, leukemia, and adrenal gland cancer cells. As shown in Table S2, many compounds showed strong antiproliferation activity against most of the cell lines. More than 20 compounds showed more than 90% inhibition of all of the tested cell lines at 20 *μ*M. Notably, five caged xanthones exhibited strong cytotoxicity on all of the cell lines, which are highlighted in gray in Table S2. The structures of these compounds were elucidated in Figure 1. We then examined the IC50 values of these xanthones against thirteen cancer cell lines, including lung, brain, colon, vulva, skin, and bone cancer cells. From the data shown in Table 1, we found that all of the xanthone compounds exhibited high potency on all of the cancer lines, with IC50s less than 5 *μ*M (except on H1573 cells). We noticed that the caged xanthones had the lowest IC50s against NCI-H1650 cells. It is notable that NCI-H1650 cells contained high STAT3 activity. In addition, gambogic acid, another caged xanthone, was able to inhibit STAT3 activation upon IL-6 treatment in multiple cancer cell lines [16]. We then intended to investigate if these caged xanthones were specific essential inhibitors for STAT signaling pathway. Therefore, NCI-H1650 cells were chosen for further studies of the effects of these compounds on viability and colony formation. For effects on viability, Figure 2(a) showed that 5 caged xanthone compounds inhibited the growth of NCI-H1650 cells in a time-dependent manner, which was assessed by a trypan blue exclusion assay. To measure the IC50s of these compounds, cell proliferation curves were generated using the CellTiter-Glo kit. NCI-H1650 cells were treated for 72 hr with compounds at concentrations ranging from 20 *μ*M to 0.009 *μ*M in serial 3-fold dilutions. The IC50s were less than 2 *μ*M for all five compounds. In addition, these caged xanthones significantly suppressed the clonogenic activity of the cells (Figure 2(b)). Particularly, GB39 and GB40 were more potent than other three compounds in both cell growth and colony formation assays.

### 3.2. The Five Caged Xanthones Induce Caspase-Dependent Apoptosis and Cell Cycle Arrest in NCI-H1650 Cells

**Figure 3:**
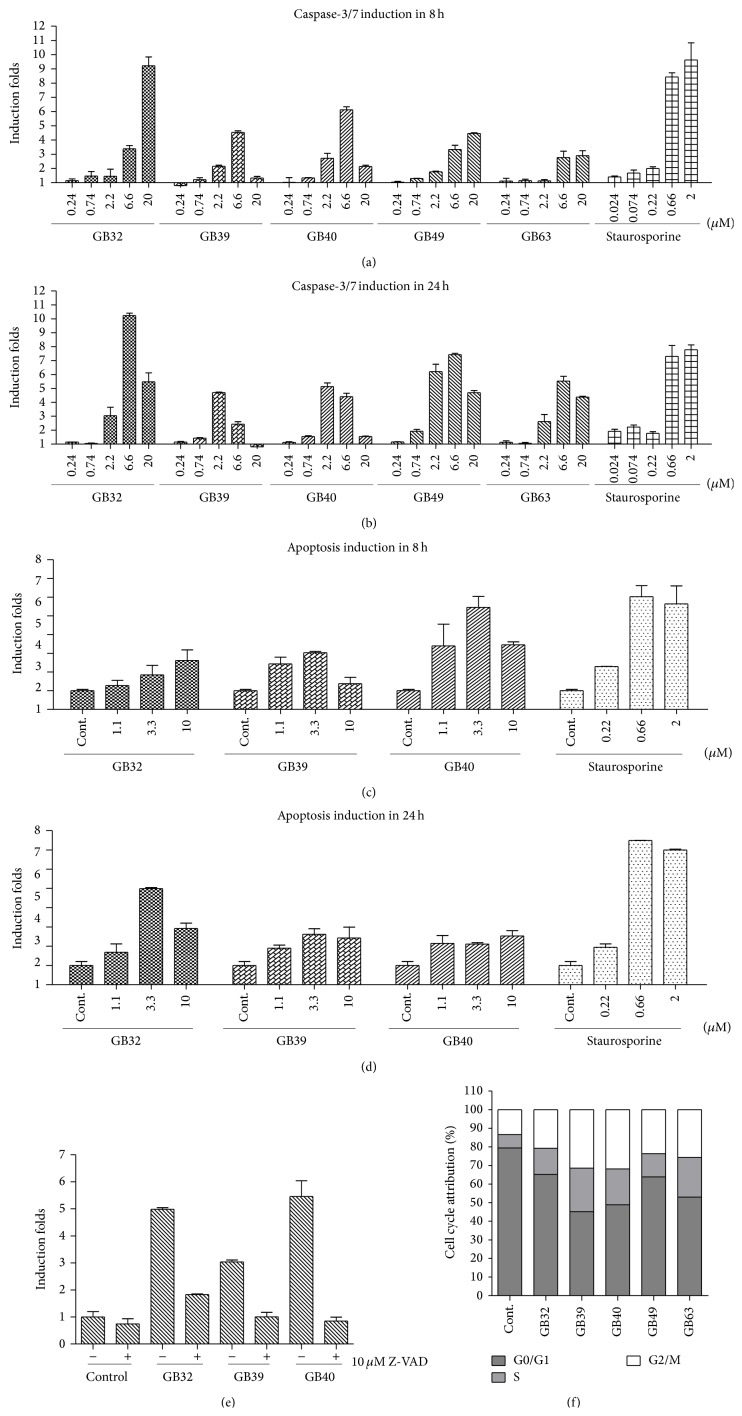
Caspase-3/7 induction and apoptosis induction by cell death detection in NCI-H1650 cells after compound treatment. (a) and (b) Caspase-3/7 activity induction was evaluated after 8 and 24 hr of treatment with the five compounds (GB32, GB39, GB40, GB49, and GB63) using Caspase-Glo 3/7 kit from Promega. Bars on the graph represent the mean fold induction relative to the DMSO control. (c) and (d) Apoptosis induction of cell lysate was assessed by oligonucleosomal fragmentation after 8-hour and 24-hour incubation with treatment compounds (GB32, GB39, and GB40). Data are shown relative to DMSO controls set at 1.0 ± SEM. (e) Apoptosis induction in NCI-H1650 cells by GB32, GB39, and GB40 was assessed ± the caspase inhibitor Z-VAD (10 *μ*mol/L). The maximal apoptosis induction for each compound was chosen for the assessment of apoptosis reversal by Z-VAD. Data are shown relative to DMSO controls set at 1.0 ± SEM. (f) Compounds induced accumulation of G2 phase cells. NCI-H1650 cells were treated with 2.5 *μ*M of each compound for 16 h. The cells were harvested, fixed in 70% EtOH, and stained with PI. The cell cycle was detected by FACS.

To determine whether the cell growth inhibition in response to the caged xanthones was mediated by apoptosis and was caspase dependent, NCI-H1650 cells were treated with 20, 6.66, 2.22, 0.74, and 0.24 *μ*M of the five caged xanthones for 8 and 24 h. As shown in Figures 3(a) and 3(b), caspase-3/7 activity was activated in a dose-dependent manner. Caged xanthones significantly increased caspase-3/7 activity more than 10-fold at a certain concentration and time point. Because GB32, GB39, and GB40 showed more potency after 8-hour treatment, we chose these three compounds for confirmation of their apoptotic effect using an ELISA-based assay (Cell Death Analysis kit, Roche). This photometric assay measured the histone-associated DNA fragments that are released during apoptotic cell death. We found that the majority (>90%) of NCI-H1650 cells treated with GB39 or GB40 underwent rapid apoptosis after 8 h, but GB32 showed significant apoptosis after 24 h (Figures 3(c) and 3(d)). To further confirm the apoptosis induction by these compounds, we applied a pan-caspase inhibitor (Z-VAD) to investigate whether Z-VAD could rescue the cells from death. As shown in Figure 3(e), Z-VAD cotreatment completely suppressed GB32-, GB39-, and GB40-induced oligonucleosomal fragmentation, suggesting that these caged xanthones caused caspase-dependent cell death. In addition, cell necrosis was not detected at any of the tested concentrations (data not shown), indicating that apoptosis is the predominant mechanism of cell death. In the above experiments, the treatment for 24 h sometimes caused the decreased induction of caspase-3/7 and apoptosis (Figures 3(b) and 3(d)). It might due to the apoptosis occurring in the early time point so that there was less amount of cells in the 24 h.

To examine whether the inhibitory effect on proliferation is caused by cell cycle arrest, we used propidium iodide (PI) staining and flow cytometry to analyze the cell cycle. NCI-H1650 cells were treated with the five compounds for 24 hours at 2 *μ*M. A persistent accumulation of S or G2/M phase cells was observed, as shown in Figure 3(f). Taken together, our results suggested that these caged xanthones inhibited cell growth by inducing caspase-dependent apoptosis and cell cycle arrest.

### 3.3. The Caged Xanthones Activate Proapoptotic BH3-Only Genes

Global analysis of gene expression has yielded numerous novel transcriptional target genes, including some with clear proapoptotic properties, such as the BH3-only proteins BBC3/PUMA [17, 18], PMAIP1/NOXA [19], and CDIP [20].

**Figure 4:**
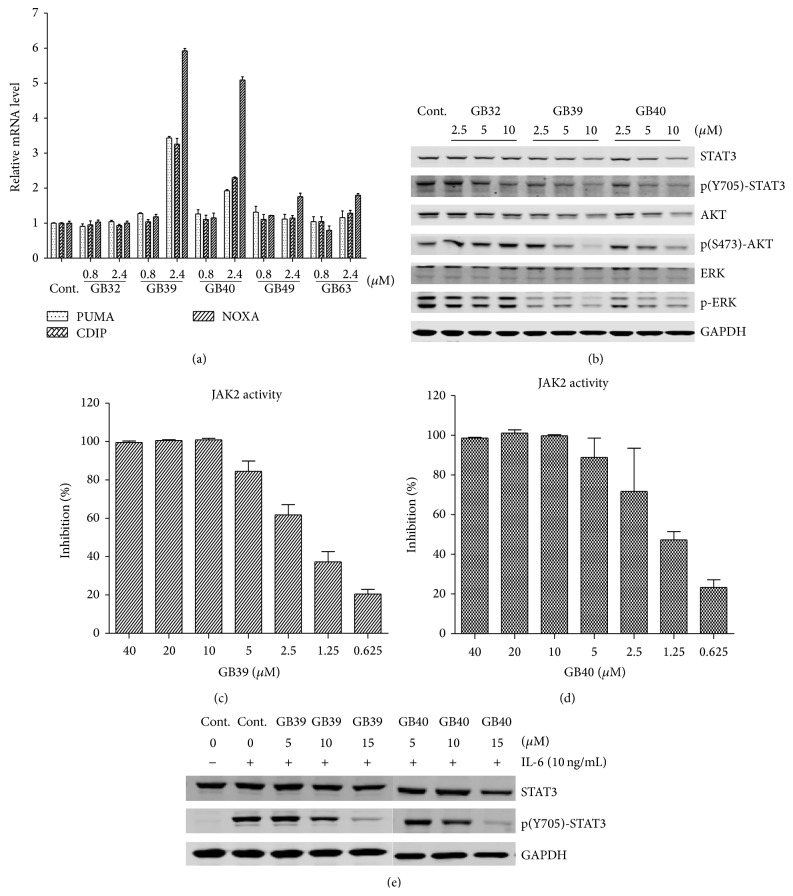
Assessing the mechanism of apoptosis induction for the compounds. (a) mRNA expression level of proapoptotic genes after treatment. NCI-H1650 cells were treated with different concentrations of each compound for 24 hr. Cells were harvested for RNA extraction. Q-PCR was performed by the TaqMan method on total RNA using primers specific for the PUMA, NOXA, and CDIP genes. (b) Immunoblot demonstrating the effect of GB32, GB39, and GB40 treatment on ERK, AKT, and STAT3 in NCI-H1650 cells. Treatment compounds were incubated for 8 h, and then whole cell lysates were processed for western blot analysis. Immunoblots were probed with the indicated antibodies. GAPDH served as a loading control. (c) GB39 significantly inhibits JAK2 of JAK family in a dose-dependent manner. After compound and substrates were added, the enzyme reaction was initiated by adding JAK2 enzyme and incubated for 2 hours. The reaction was then stopped, and the signal was detected using a Caliper LabChip 2000 plate reader. (d) GB40 significantly inhibits JAK2 of JAK family in a dose-dependent manner. After compound and substrates were added, the enzyme reaction was initiated by adding JAK2 enzyme and incubating for 2 hours. The reaction was then stopped, and the signal was detected using a Caliper LabChip 2000 plate reader. (e) HepG2 cells were pretreated with GB39 and GB40 for 2 h before stimulation by IL-6 (10 ng/mL) for 15 min. Whole cell lysates were processed for western blot analysis, and immunoblots were probed with the indicated antibodies. GAPDH served as a loading control.

Quantitative real-time reverse transcription-PCR confirmed that these proapoptotic genes (PUMA, NOXA, and CDIP) were upregulated preferentially in GB39- and GB40-treated NCI-H1650 cells. As shown in Figure 4(a), there was greater than 5-fold induction of NOXA for GB39 and GB40 treatment, 3-fold induction of PUMA and CIDP for GB39, and 2-fold induction of PUMA and CIDP following 24-hour treatment of NCI-H1650 cells with GB40. On the contrary, treatment with GB32, GB49, or GB63 had no effect on these three proapoptotic genes in NCI-H1650 cells.

### 3.4. Caged Xanthones Inhibit the JAK-STAT3 Signaling Pathway in Cancer Cells

Several studies have demonstrated that STAT3 is required for efficient cellular transformation by oncogenes including Ras, v-Src, SV40 T antigen, and EGFR, further validating the importance of STAT3 in cancer biology [21–25]. STAT3 is both a cytoplasmic signaling molecule and a nuclear transcription factor that is activated by the phosphorylation of specific tyrosine residue in its carboxyl-terminus by JAK kinases in response to cytokines, including IL-6, IFN, epidermal growth factor, and FGF [26, 27]. In the nucleus, STAT3 regulates the expression of the proteins that regulate mitochondria-mediated apoptosis, such as Bcl-2, Mcl-1, and cIAP [28]. The STAT3 tyrosine phosphorylation in malignancies is activated via increased/sustained IL-6 (family)/gp130 signaling [29, 30]. The other “oncogenic” pathways, such as those mediated by EGFR, HER2, Ras, and Rho, can also result in increased IL-6 production and subsequent STAT3 activation [31–33]. STAT3 is activated in lung adenocarcinomas through a mutant EGFR regulating the expression of the IL-6 cytokine, which, in turn, activates the gp130/JAK pathway. NCI-H1650 is a lung adenocarcinoma cell, in which a mutant EGFR regulates the expression of the IL-6 cytokine and leads to the activation of JAK/STAT3 pathway.

NCI-H1650 cancer cells contain an EGFR mutant and a constitutively high level of STAT3 phosphorylation. We then investigated whether the caged xanthones induced apoptosis via modulation of the JAK-STAT3 signaling pathway [34]. We evaluated the protein level of STAT3 and the phosphorylation level of STAT3 at Tyr705.  Figure 4(b) shows that GB39 and GB40 inhibited STAT3 expression and its phosphorylation in a dose-dependent manner. We further examined the other components involved in the STAT3 signaling pathway. Phosphorylation of ERK and AKT was also inhibited by GB39 and GB40, which confirmed that these two caged xanthones could regulate the STAT3 pathway.

STAT3 has been reported to be activated by soluble tyrosine kinases of the JAK family, and the phosphorylation of STAT3 at residue Tyr705 is mediated by receptor-associated tyrosine kinases, such as JAKs. We therefore examined the activity of these compounds against commercial, recombinant JAK enzymes (TYK2, JAK1, JAK2, and JAK3) using an enzymatic test. As shown in Table 2, GB32, GB39, and GB40 exhibited different inhibitory activities on the JAK family enzymes. GB39 and GB40 showed stronger inhibition and lower IC50 against JAK2/JAK3. Particularly, these two compounds are the more potent against JAK2 (IC50 value of approximately 1 *μ*M) than against the other three Jak kinases. A 10-fold difference in IC50s was observed between JAK2 and Tyk2 for GB39 and GB40. Figures 4(c) and 4(d) show the dose-dependent inhibition of JAK2 activity by GB39 and GB40, respectively. These data suggested that GB39 and GB40 directly inhibited the catalytic activity, especially that of JAK2, and the inhibition of JAK family enzymes leads to the blockade of phosphorylation of STAT3. Meanwhile, GB32 only showed minor inhibition of JAK2/JAK3 and had no effect on TYK2/JAK1. In addition, we examined the effects of GB39 and GB40 on IL-6-stimulated STAT3 activation. As shown in Figure 4(e), these two compounds efficiently suppressed the phosphorylation of STAT3 upon IL-6 stimulation in HepG2 cells. Our study indicated that GB39 and GB40 were identified as potent inhibitors of the JAK/STAT3 signaling pathway. In addition, the direct targets of GB39 and GB40 were discovered to be JAK2 and JAK3. GB39 and GB40 significantly inhibited JAK2/ JAK3 kinase activities, and the Y705 site in STAT3 was not phosphorylated after GB39 and GB40 treatment. Furthermore, the inhibitory effects of GB39 and GB40 on JAK kinase activities were JAK2/3-specific. GB39 and GB40 had minor inhibitory effects on JAK1/Tyk2 (Table 2), which indicated that GB39 and GB40 have the potential to be JAK/STAT3 signaling pathway-specific inhibitors. Interestingly, we also detected the similar inhibitory effects on STAT3 phosphorylation of gambogic acid, a caged xanthone, on both NCI-H1650 and HepG2 cells (Supplement Figures S1(A) and S1(B)). Taken together, our results indicated that the caged xanthones family could inhibit STAT3 activation through suppressing JAKs activities. Therefore, it will be promising to develop specific JAKs inhibitors from the large amount of natural caged xanthones. Alternatively, the caged xanthones might also be used to combine treatment with other anticancer agents to show synergetic antitumor effects.

## 4. Conclusions 

Overall, our study suggested that caged xanthones exhibited strong anticancer activity on a panel of cancer cell lines, especially those whose survival and growth are dependent on constitutively active STAT3 signaling. We discovered that GB39 and GB40, two natural caged xanthones, were potent inhibitors of the JAK/STAT3 signaling pathway. GB39 and GB40 suppressed constitutive STAT3 activation by direct inhibition of JAK kinase activity. Finally, our results suggested that two caged xanthones were JAK2 kinase inhibitors and were potentially worth developing as novel anticancer drugs. Future studies will focus on evaluating the antitumor activities of GB39 and GB40 in animal models.

## Supplementary Material

To better understand the anti-proliferation activities of the 64 compounds isolated from *Garcinia* plants in our lab, it is necessary to perform a cell proliferation assay using multiple cell lines to establish their cytotoxicity. The source and structure information of these compounds were shown in Table S1. The cytotoxicity data of these compounds at 20 µM was obtained by cell proliferation assay and shown in Table S2.
To better illustrate these five caged xanthones are specific essential inhibitors for STAT signaling pathway, Gambogic acid, another caged xanthones, inhibits STAT3 activation upon autocrined IL-6 by NCI-H1650 cell or IL-6 treatment in HepG2 cell were elaborated in Figure S1 by western blot.

## Figures and Tables

**Table 1:** The IC50s for the chosen 5 compounds and reference compound (staurosporine) against the 13 cancer cell lines and the control HuVec cell line.

Cell	GB32	GB39	GB40	GB49	GB63	Staurosporine
NCI-H838	3.15 ± 0.04	1.5 ± 0.07	1.68 ± 0.19	2.72 ± 0.15	2.65 ± 0.2	0.011 ± 0.004
A549	2.88 ± 0.06	3.04 ± 0.1	2.45 ± 0.06	3.46 ± 0.2	3.77 ± 0.06	0.008 ± 0.002
NCI-H1573	4.36 ± 0.15	6.08 ± 0.21	5.99 ± 0.19	7.89 ± 0.14	7.92 ± 0.29	0.039 ± 0.01
NCI-H1975	1.58 ± 0.12	1.25 ± 0.14	0.96 ± 0.05	1.69 ± 0.15	1.71 ± 0.22	0.002 ± 0.001
NCI-H1993	2.47 ± 0.11	1.03 ± 0.13	1.29 ± 0.05	2.55 ± 0.14	2.15 ± 0.1	0.021 ± 0.007
NCI-H1650	**1.09 ± 0.15**	**0.95 ± 0.03**	**0.62 ± 0.03**	**0.97 ± 0.04**	**1.43 ± 0.09**	**0.022 ± 0.004**
CHP212	1.45 ± 0.08	1.2 ± 0.08	1.18 ± 0.05	1.43 ± 0.03	3.51 ± 0.08	0.004 ± 0.001
U118MG	1.19 ± 0.07	1.15 ± 0.09	1.03 ± 0.15	1.22 ± 0.05	3.05 ± 0.1	0.003 ± 0.001
SW48	3.84 ± 0.09	1.44 ± 0.06	1.47 ± 0.1	2.97 ± 0.06	4.02 ± 0.15	0.025 ± 0.003
LnCap	1.8 ± 0.14	0.84 ± 0.08	0.79 ± 0.12	1.91 ± 0.06	2.17 ± 0.21	0.061 ± 0.009
SW954	2.43 ± 0.17	0.94 ± 0.04	1.02 ± 0.1	1.88 ± 0.1	2.58 ± 0.16	0.006 ± 0.001
SK-MEL-28	1.51 ± 0.07	0.82 ± 0.11	0.73 ± 0.21	1.51 ± 0.12	1.75 ± 0.16	0.37 ± 0.058
SW1353	1.41 ± 0.05	0.76 ± 0.03	0.83 ± 0.05	1.29 ± 0.05	2.48 ± 0.11	0.004 ± 0.001
HuVec	1.63 ± 0.06	0.89 ± 0.09	0.68 ± 0.01	1.47 ± 0.07	1.9 ± 0.03	0.004 ± 0.002

**Table 2:** The IC50s for GB32, GB39, and GB40 against the JAK1, JAK2, JAK3, and Tyk2 enzymes.

IC50 [*μ*M]	GB32	GB39	GB40
JAK1	>40	12.2 ± 0.3	14.2 ± 0.4
JAK2	37.3 ± 6.9	1.7 ± 0.1	1.4 ± 0.1
JAK3	17.5 ± 1.5	3.8 ± 0.3	3.2 ± 0.2
Tyk2	>40	13 ± 1.6	12.9 ± 0.9

## Abbreviations

GA: Gambogic acid
IL-6: Interleukin 6
PI: Propidium iodide
STAT: Signal transducer of activator of transcription
PPAPs: Polycyclic polyprenylated acylphloroglucinols.
