# Supplementary material for: Screening Active Compounds from Garcinia Species Native to China Reveals Novel Compounds Targeting the STAT/JAK Signaling Pathway
Source: Biomed Res Int. 2015 May 18;2015:910453. doi: 10.1155/2015/910453 (PMC4450297; doi:10.1155/2015/910453)
Supplement: Supplementary file 1 — To better understand the anti-proliferation activities of the 64 compounds isolated from Garcinia plants in our lab, it is necessary to perform a cell proliferation assay using multiple cell lines to establish their cytotoxicity. The source and structure information of these compounds were shown in Table S1. The cytotoxicity data of these compounds at 20 µM was obtained by cell proliferation assay and shown in Table S2. To better illustrate these five caged xanthones are specific essential inhibitors for STAT signaling pathway, Gambogic acid, another caged xanthones, inhibits STAT3 activation upon autocrined IL-6 by NCI-H1650 cell or IL-6 treatment in HepG2 cell were elaborated in Figure S1 by western blot. [file 910453.f1.pdf]

Table S1: Source and structure for 64 compounds from Gancinia plant

Compound	Name	Source	Structure
bn-1	Garcinone E	*Garcinia xishuanbannaensis*	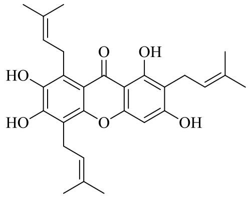
bn-10	1,3,6,7-tetrahydroxy-8-(3-methylbut- 2-enyl)xanthone	*Garcinia xishuanbannaensis*	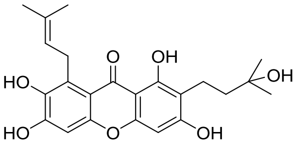
bn-16	Garcinone C	*Garcinia xishuanbannaensis*	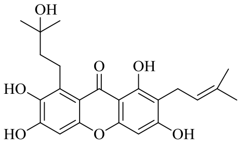
bn-17	Bannaxanthone B	*Garcinia xishuanbannaensis*	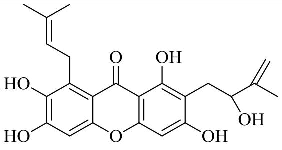
bn-2	Allanxanthone C	*Garcinia xishuanbannaensis*	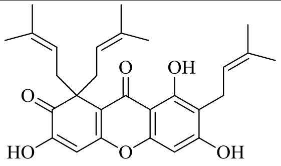
bn-21	Bannaxanthone G	*Garcinia xishuanbannaensis*	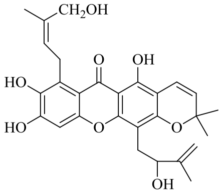
bn-3	Bannaxanthone E	*Garcinia xishuanbannaensis*	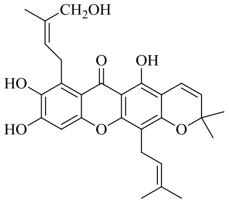
bn-4	Bannaxanthone D	*Garcinia xishuanbannaensis*	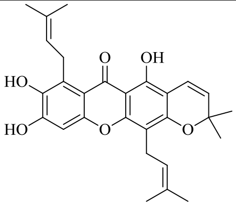

C-6	1,5,6-Trihydroxy-2-prenyl-6',6'-dimethyl-2*H*-pyrano(2',3':3,4)xanthone	*Garcinia cowa*	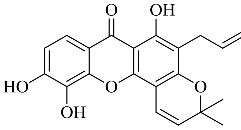
GB11	*S*-gambogic acid	*Garcinia hanburyi*	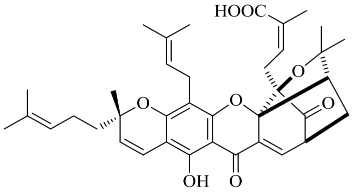
GB14	*R/S*-isogambogic acid	*Garcinia hanburyi*	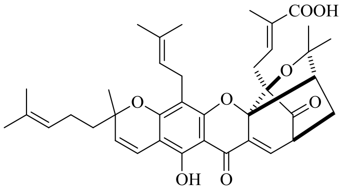
GB16	Gambogenic acid	*Garcinia hanburyi*	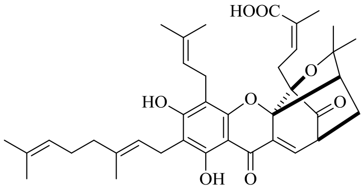
GB18	Isomorellinol	*Garcinia hanburyi*	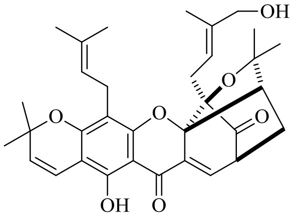
GB19	Gambogoic acid B	*Garcinia hanburyi*	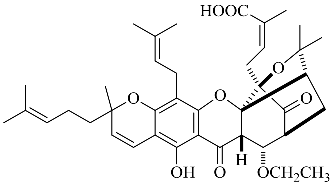
GB27	Isomorellic acid	*Garcinia hanburyi*	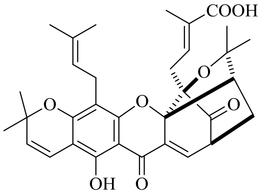
GB32	Desoxygambogenin	*Garcinia hanburyi*	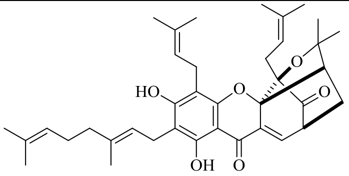

GB35	Forbesione	*Garcinia hanburyi*	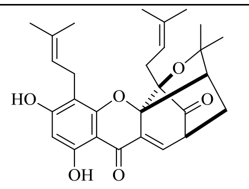
GB39	*S*-33-hydroxygambogic acid	*Garcinia hanburyi*	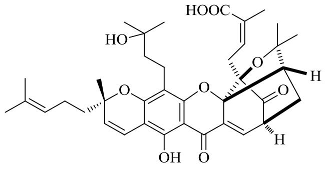
GB40	*S*-35-hydroxygambogic acid	*Garcinia hanburyi*	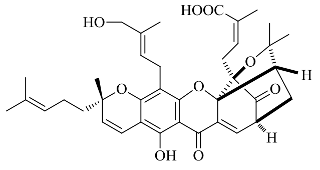
GB49	Isogambogenin	*Garcinia hanburyi*	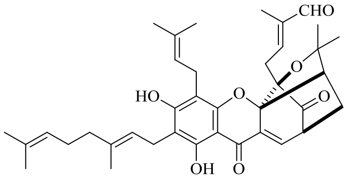
GB63	Isogambogenic acid	*Garcinia hanburyi*	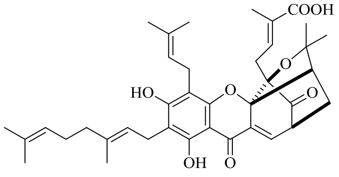
GB8	*R/S*-gambogic acid	*Garcinia hanburyi*	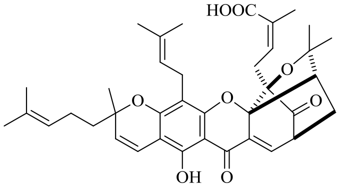
Ge-1	1,3,7-trihydroxy-2-(3-methylbut-2-enyl)-xanthone	*Garcinia esculenta*	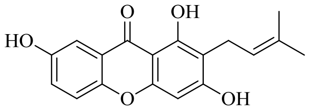
Ge-10	Toxyloxanthone B	*Garcinia esculenta*	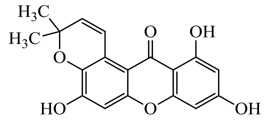
Ge-15	1,3,5,7-tetrahydroxy-8-isoprenyl xanthone	*Garcinia esculenta*	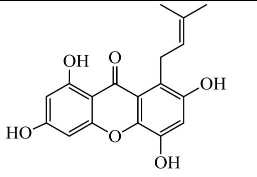

Ge-17	1,3,6,7-tetrahydroxyxanthone	*Garcinia esculenta*	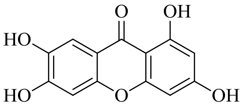
Ge23	Guttiferone F	*Garcinia esculenta*	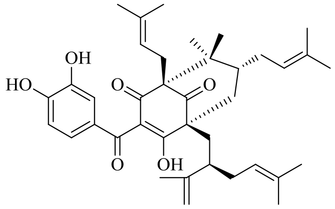
Ge27	Garcimultiflorone E	*Garcinia esculenta*	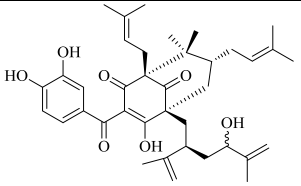
Ge-3	Griffipavixanthone	*Garcinia esculenta*	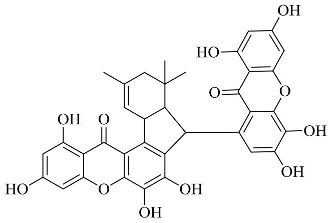
Ge-6	Cambogin	*Garcinia esculenta*	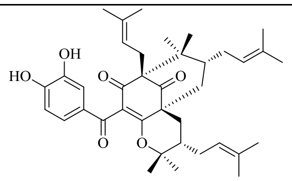
Ge6-3	Garciniagifolone A	*Garcinia esculenta*	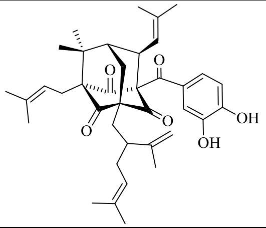
Ge9	*γ*-mangostin	*Garcinia esculenta*	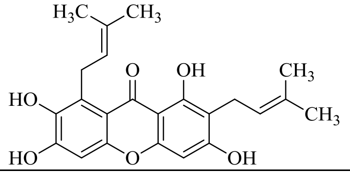
gl-11-1	1,6,7-trihydroxy-6,6'-dimethyl-2H-pyrano(2',3',:3,2)-4-(3-methylbut-2-enyl)xanthone	*Garcinia lancilimba*	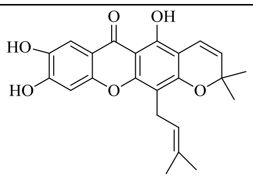

gl-12	Nigrolineaxanthone V	*Garcinia lancilimba*	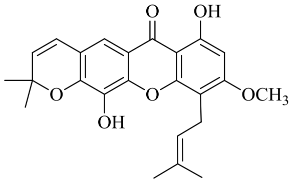
gl-14	30-epicambogin	*Garcinia lancilimba*	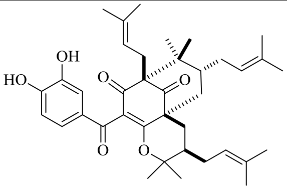
gl-16	1,3,5-trihydroxy- 6-methoxy-7-prenylxanthone	*Garcinia lancilimba*	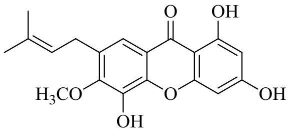
gl-17	1,5,6-trihydroxy- 3-methoxy- 4-prenylxanthone	*Garcinia lancilimba*	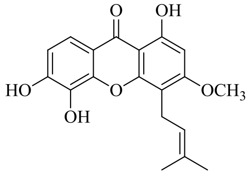
gl-18	Isojacareubin	*Garcinia lancilimba*	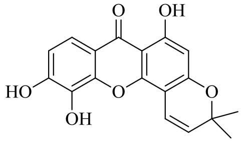
gl-20	1,7-dihydroxyxanthone	*Garcinia lancilimba*	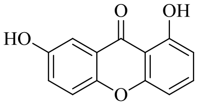
gl-23	1,5,6-trihydroxyxanthone	*Garcinia lancilimba*	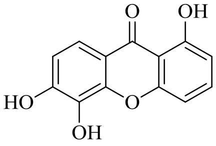
gl-25	Macurin	*Garcinia lancilimba*	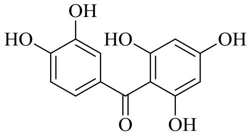
gl-34	Taxfolin	*Garcinia lancilimba*	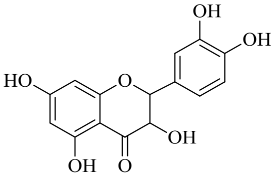

gl-6	1,3-dihydroxy- 5,6-dimethoxy- 7-prenylxanthone	*Garcinia lancilimba*	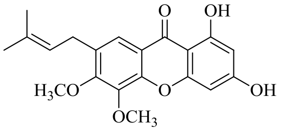
gl-7	(+) guttiferone B	*Garcinia lancilimba*	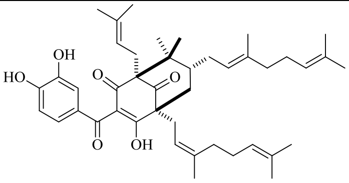
gl-9	Dulxanthone B	*Garcinia lancilimba*	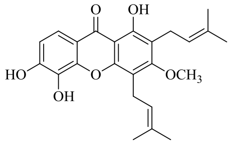
gly-2	(2*S*)-eriodictyol-7-*O*-*β*-D-glucopyranosiduronic acid	*Garcinia lancilimba*	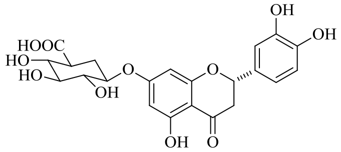
gly-3	(2*S*)-eriodictyol 7-*O*-*β*-D-glucuronide ethyl ester	*Garcinia lancilimba*	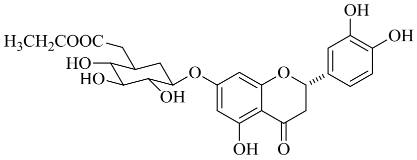
gly-5	(2S)-eriodictyol 7-O-β-D-glucuronide methyl ester	*Garcinia lancilimba*	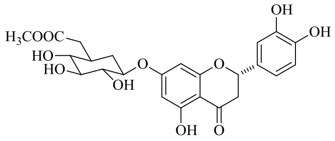
Go-11	1,3,5-trihydroxy-13,13-dimethyl-2H-pyran(7,6-b) xanthone	*Garcinia oblongifolia*	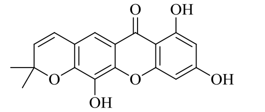
Go-14	Leiaxanthone	*Garcinia oblongifolia*	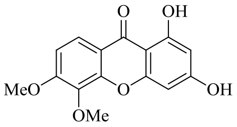
Go-15	Parvifolixanthone B	*Garcinia oblongifolia*	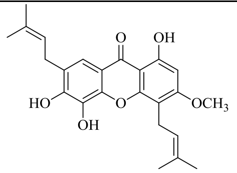
Go-16	Rheediaxanthone A	*Garcinia oblongifolia*	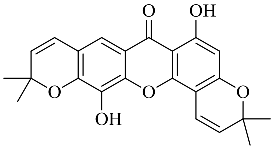

Go-23	Oblongixanthone C	*Garcinia oblongifolia*	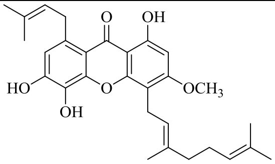
Go-24	Oblongifolin C	*Garcinia oblongifolia*	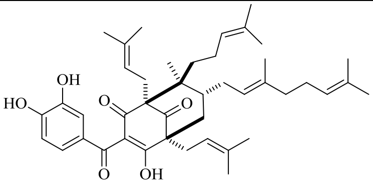
Go-24a	Oblongifolin D	*Garcinia oblongifolia*	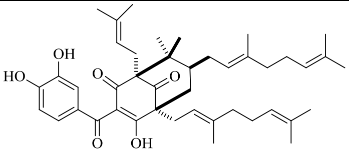
Go-9	Nigrolineaxanthone T	*Garcinia oblongifolia*	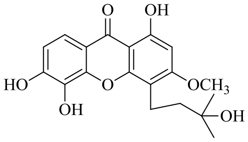
Gof1	Garcihombronone D	*Garcinia oblongifolia*	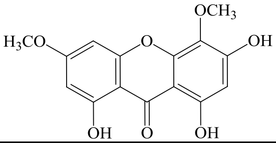
Gof3	Euxanthone	*Garcinia oblongifolia*	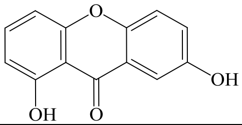
Gxc-3	Xanthone V1	*Garcinia cowa*	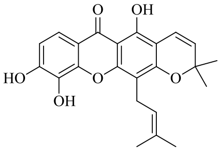
Gxy-11	Oblongifolin A	*Garcinia yunnanensis*	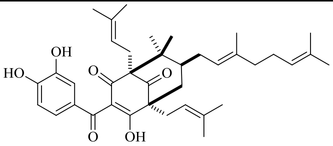
Gxy-4	Guttiferone K	*Garcinia yunnanensis*	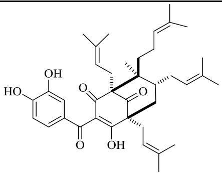
N-7	Nujiangexanthone A	*Garcinia nujiangensis*	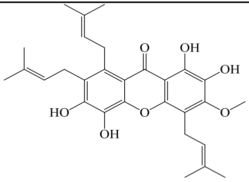

WZ-4	Morelloflavone	*Garcinia yunnanensis*	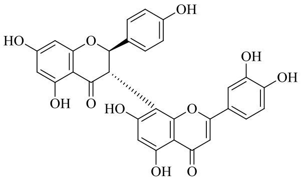
WZ-6	Apigenin-7-*O*-*β*-D-glucuronide methyl ester	*Garcinia yunnanensis*	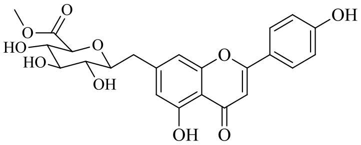

Table S2 : Percent Inhibition data of 64 compounds at the 20 micromolar against 36 cancer cell lines in pre-screen study.

Cell Type\ Compound		bn-1	bn-10	bn-16	bn-17	bn-2	bn-21	bn-3	bn-4	C-6	GB11	GB14	GB16	GB18	GB19	GB27	GB32	GB35	GB39	GB40	GB49	GB63	GB8	Ge-1	Ge-10	Ge-15	Ge-17	Ge23	Ge27	Ge-3	Ge-6	Ge6-3	Ge9
Lung	A498	98	25	65	54	34	60	96	84	10	98	97	100	93	99	88	98	93	99	98	88	98	99	54	33	46	41	45	53	78	97	98	98
	ChaGo-K-1	99	18	91	73	75	95	97	93	7	98	98	99	99	99	98	99	99	100	98	99	100	99	44	26	36	22	55	77	97	98	98	97
	MSTO-211-H	98	4	93	70	72	90	98	94	1	98	97	100	99	99	99	99	99	100	98	99	99	100	44	48	34	74	50	76	96	99	93	98
	NCI-H1623	91	-10	67	20	49	72	95	80	-13	98	98	99	99	99	98	99	100	100	99	99	99	99	9	12	-16	2	-2	10	94	99	98	91
	NCI-H1694	100	28	99	96	99	100	99	97	19	99	98	100	99	99	99	100	100	100	99	100	100	100	49	32	68	99	50	95	99	100	99	99
	NCI-H1770	93	0	87	12	90	94	98	75	5	98	97	100	99	99	99	99	100	100	99	99	99	100	13	3	-4	72	8	45	92	99	99	98
	NCI-H2347	79	9	73	23	40	75	89	53	7	98	97	98	97	98	93	98	97	99	98	98	98	99	32	22	10	4	29	54	60	71	74	89
	NCI-H2405	93	-8	73	7	40	80	96	92	-5	98	97	99	99	99	98	99	99	100	99	99	99	99	7	-4	-11	-5	-5	11	86	99	98	96
	NCI-H28	95	12	71	69	52	73	95	85	12	99	97	99	98	99	90	99	93	99	98	97	99	99	23	10	27	9	38	55	79	91	89	97
	NCI-H524	100	-20	98	-19	97	100	99	98	-61	97	98	98	100	99	99	99	100	100	99	100	98	98	42	38	-54	47	-7	97	99	99	98	97
Breast	BT-474	87	-3	83	27	22	88	90	83	-18	98	98	99	99	99	98	99	99	100	99	99	99	99	30	-25	9	-7	3	25	94	91	84	97
	BT-549	98	-4	74	34	54	70	97	96	-6	99	98	99	99	99	98	99	100	100	99	99	99	100	20	7	6	40	9	49	95	95	93	97
	HCC1395	100	8	94	83	84	95	98	96	14	98	98	99	99	99	99	99	100	100	98	99	99	99	55	45	45	38	57	85	99	99	98	98
	HCC1569	96	16	81	51	63	76	97	92	7	99	98	99	99	99	99	99	100	100	99	99	99	100	52	21	24	16	9	62	98	98	95	97
	HCC1599	98	77	94	84	79	97	98	97	52	99	98	100	99	99	99	100	100	100	99	99	99	100	54	30	78	79	23	85	96	98	93	95
	HCC1937	97	32	80	58	75	92	98	89	21	98	98	100	98	99	99	99	100	100	99	99	100	100	11	19	43	65	44	74	97	99	96	98
	T47D	82	4	62	45	54	64	96	80	6	99	98	99	99	99	99	99	100	100	99	99	99	100	45	26	43	48	37	59	91	92	91	94
	ZR-75-30	99	77	94	81	86	99	99	81	64	99	99	100	99	99	99	99	100	100	99	100	99	99	81	65	85	6	74	89	99	100	99	99
Urinary bladder	HT-1197	97	8	85	44	38	91	98	89	20	98	98	99	99	99	95	99	96	100	99	96	99	99	50	44	31	16	43	53	88	90	90	97
	J82	98	29	85	52	43	88	98	94	13	98	97	99	99	99	98	99	99	100	99	98	99	99	38	21	44	47	37	54	93	97	83	97
	TCCSUP	97	33	88	79	75	84	96	92	11	99	98	100	99	99	99	99	100	100	99	99	99	100	28	28	49	61	43	75	94	97	99	99
Uterus	Colo 205	98	19	97	57	69	100	99	93	3	98	98	99	99	99	99	99	100	100	99	100	99	99	29	16	21	3	47	93	99	97	98	98
	RL95	99	15	69	45	70	88	98	96	9	99	97	99	99	99	98	99	100	100	99	99	99	100	37	9	32	53	10	75	98	98	96	98
	HEC-1-A	87	22	61	46	51	71	88	76	1	99	97	99	99	99	99	99	100	100	99	99	99	100	26	25	25	51	23	57	73	83	85	95
Brain	CCF-STTG1	89	8	65	43	52	68	93	86	16	98	98	99	99	99	99	99	100	100	99	99	99	99	43	18	17	15	30	42	94	87	92	99
	M059J	95	9	78	48	51	94	97	90	-21	98	97	99	99	99	99	99	100	100	99	99	99	100	24	19	23	63	4	65	96	93	95	-17
	T98G	100	21	91	94	98	77	99	97	11	99	98	99	99	99	99	99	100	100	99	99	99	100	46	36	52	54	39	82	97	99	97	98
Liver	Bel-7402	98	7	91	57	64	93	98	94	5	98	97	99	99	99	97	99	99	100	99	98	99	99	19	5	16	7	44	80	97	98	98	99
	C3A	90	20	43	39	39	62	78	63	17	98	98	99	99	99	98	99	98	99	98	97	99	99	32	21	32	33	24	34	49	88	72	81
Pancreas	HPAF-II	90	-2	75	14	28	84	92	73	-10	98	97	98	99	98	98	99	100	100	99	98	99	99	28	20	16	13	21	38	61	88	69	96
	Panc 03.27	90	-1	86	44	48	95	92	88	21	99	97	99	99	99	97	99	99	100	99	98	99	99	43	26	20	20	24	55	93	77	64	97
Stomach	HGC-27	99	30	77	62	53	84	98	86	18	98	98	100	99	99	98	99	100	100	99	98	99	100	54	31	42	55	45	66	81	99	98	97
Colon	CW-2	99	25	95	68	72	99	99	97	5	98	98	99	99	99	99	99	100	100	99	99	99	99	62	29	64	25	49	72	98	99	99	99
Kidney	A704	98	-11	62	19	4	64	98	94	-8	98	97	99	99	99	98	99	99	99	98	98	99	99	34	23	-3	1	4	25	80	99	73	96
Leukemia	C8166	99	23	88	71	86	90	98	96	12	99	97	100	99	99	99	99	99	100	99	99	100	100	9	11	20	62	39	81	96	98	99	99
Adrenal gland	SW13	97	11	96	52	15	92	98	94	1	98	97	99	99	99	99	99	100	100	98	99	99	99	57	27	16	8	7	54	99	97	99	-3

Cell Type\ Compound		gl-11-1	gl-12	gl-14	gl-16	gl-17	gl-18	gl-20	gl-23	gl-25	gl-34	gl-6	gl-7	gl-9	gly-2	gly-3	gly-5	Go-11	Go-14	Go-15	Go-16	Go-23	Go-24	Go-24a	Go-9	Gof1	Gof3	Gxc-3	Gxy-11	Gxy-4	N-7	WZ-4	WZ-6
Lung	A498	-2	11	95	34	75	74	38	3	-10	2	47	51	86	-7	-4	-12	37	-2	78	12	94	81	43	64	6	39	90	65	74	13	4	13
	ChaGo-K-1	-17	38	96	57	87	91	57	-22	-26	-3	59	94	97	-33	-13	-10	75	-16	11	9	94	97	94	94	-21	12	99	89	97	4	-14	-16
	MSTO-211-H	0	-5	97	61	79	85	59	-25	-30	-9	16	89	98	7	-3	-6	66	11	69	34	95	97	91	92	14	36	98	82	95	-1	6	6
	NCI-H1623	16	5	97	18	41	77	-13	0	0	-7	14	83	85	6	8	7	-10	-10	78	33	92	98	94	63	-13	-7	88	63	81	-8	-15	-8
	NCI-H1694	1	28	99	55	98	98	86	-9	-12	10	46	100	100	-10	1	-2	73	-3	97	5	98	99	100	100	25	29	100	100	100	25	-10	-9
	NCI-H1770	12	34	99	21	40	89	-4	-11	-10	0	23	99	93	-4	-10	-6	38	-4	75	25	98	99	98	53	11	17	96	91	98	-6	-10	-1
	NCI-H2347	5	9	74	42	86	84	48	-8	-13	2	22	63	82	-2	4	1	10	-2	51	-8	68	59	58	52	0	20	80	66	67	-18	-4	-3
	NCI-H2405	5	11	98	12	19	66	-5	-14	-9	-5	25	81	93	-2	-3	5	3	-10	50	27	98	98	88	72	-13	-13	93	70	95	-7	-14	-12
	NCI-H28	-13	2	82	41	76	77	24	-10	-9	-11	39	65	86	-21	-18	-17	33	-3	74	9	82	82	64	70	11	27	93	67	76	21	0	6
	NCI-H524	10	-2	99	56	98	96	91	-30	-74	-31	9	100	99	-15	-25	-5	83	-5	98	-35	98	98	99	100	2	18	100	5	100	-30	-18	-11
Breast	BT-474	-18	-2	94	16	89	92	50	-16	-3	-15	25	66	94	-18	-24	-15	31	-23	82	-6	70	83	68	77	-21	7	94	56	80	-21	-5	-12
	BT-549	-12	-2	97	27	65	86	46	-16	-10	-9	14	83	96	-14	-14	-12	46	-16	87	-5	96	96	78	68	-6	16	96	68	79	-14	-12	-16
	HCC1395	7	22	98	69	89	94	74	-11	-8	16	44	99	98	8	9	11	68	-8	25	68	97	97	96	98	17	35	99	95	97	22	16	21
	HCC1569	1	19	98	50	74	81	57	0	1	3	34	83	93	-6	-8	-7	41	2	86	11	97	95	80	86	2	29	94	75	84	5	-17	-18
	HCC1599	-15	52	98	67	93	92	83	18	11	42	42	90	95	-8	-18	-41	49	0	62	41	98	94	86	93	5	36	98	87	90	8	8	12
	HCC1937	-2	-7	98	50	83	85	62	-1	0	8	32	94	94	-2	3	1	21	3	85	25	96	97	95	85	9	8	95	88	95	-5	0	3
	T47D	-6	21	92	36	72	83	39	-4	-8	-6	31	65	90	-2	-4	2	35	-3	70	20	94	86	69	65	11	10	93	61	78	-9	6	4
	ZR-75-30	28	54	99	93	98	95	87	16	-3	14	90	99	99	15	13	7	62	-20	83	47	95	94	98	90	17	41	100	91	100	-11	8	-2
Urinary bladder	HT-1197	15	20	90	37	95	90	77	-11	5	10	36	90	97	-8	11	0	39	-3	51	35	96	90	91	76	1	28	98	75	82	13	-4	16
	J82	4	17	95	50	88	88	60	-2	1	3	52	92	99	-6	-7	-8	52	-8	74	23	97	96	90	80	10	20	99	66	82	17	-5	-5
	TCCSUP	11	11	97	63	83	85	67	-5	-5	4	36	91	96	-4	-1	-11	43	6	88	44	93	96	81	84	14	32	98	88	89	18	3	8
Uterus	Colo 205	6	10	98	57	97	96	3	-5	-1	1	43	99	97	-6	-6	-4	11	8	89	4	96	97	98	70	1	22	99	97	99	9	-6	4
	RL95	-8	23	97	45	87	94	67	-20	-8	3	46	85	98	2	6	6	72	-5	94	-8	97	94	89	94	-4	42	99	81	96	-16	-2	-5
	HEC-1-A	3	13	87	46	76	81	41	-6	5	-5	40	68	85	-19	-19	-16	38	-6	67	11	76	81	67	72	-4	19	88	64	76	-17	10	1
Brain	CCF-STTG1	12	16	87	25	74	81	49	-1	9	4	45	72	92	1	-4	8	44	-1	84	22	87	77	55	91	11	31	92	69	82	6	0	-3
	M059J	10	27	96	33	91	91	63	-5	-9	9	44	82	92	-1	1	6	78	0	88	31	93	84	76	93	2	53	95	77	86	10	-1	2
	T98G	-2	28	98	28	85	91	73	-2	-2	1	31	97	98	-3	-3	0	68	8	98	29	98	98	88	91	27	27	99	91	93	17	-2	4
Liver	Bel-7402	-4	5	96	17	60	78	-3	-16	-5	-9	27	87	96	-14	-5	-8	16	-8	64	15	96	97	87	69	13	45	98	83	95	15	-3	1
	C3A	2	7	82	45	89	81	58	-1	-3	-13	41	37	77	-5	-4	-1	19	-7	60	22	77	45	37	47	-11	7	80	49	54	24	-2	-1
Pancreas	HPAF-II	-2	21	94	26	89	82	19	-5	1	6	36	78	85	-1	-7	-7	18	0	72	15	82	87	78	76	-7	15	93	72	72	9	-10	-14
	Panc 03.27	-2	24	80	47	84	96	52	-5	-14	9	45	85	92	2	2	3	39	4	86	20	92	77	93	93	-3	7	94	71	76	-24	-16	-4
Stomach	HGC-27	0	34	98	58	92	92	69	-6	-3	-8	55	85	97	0	-11	-2	54	-2	98	28	97	98	96	94	0	34	98	88	92	0	-4	-8
Colon	CW-2	5	28	99	55	96	95	47	-3	-2	7	51	99	99	-2	4	1	61	5	96	17	97	99	98	99	18	39	100	99	99	22	11	12
Kidney	A704	-7	3	98	9	42	66	13	-11	-5	-13	27	31	88	-14	-17	-7	24	-13	92	17	96	59	32	56	11	23	93	49	68	1	-1	-9
Leukemia	C8166	10	25	98	77	90	92	76	-1	3	7	41	95	96	-12	-1	-5	71	2	93	13	95	96	94	79	5	29	98	95	94	10	-3	-15
Adrenal gland	SW13	-12	-2	92	41	85	96	33	-10	-10	-13	18	80	99	-14	-5	-6	24	-12	95	6	96	98	99	82	5	33	99	80	99	-14	5	1

Table S2: Cells were cultured and seeded in 96-well plates, and compounds were treated for 72 hrs. Cell proliferation was detected using a CellTiter-Glo kit

Figure S1

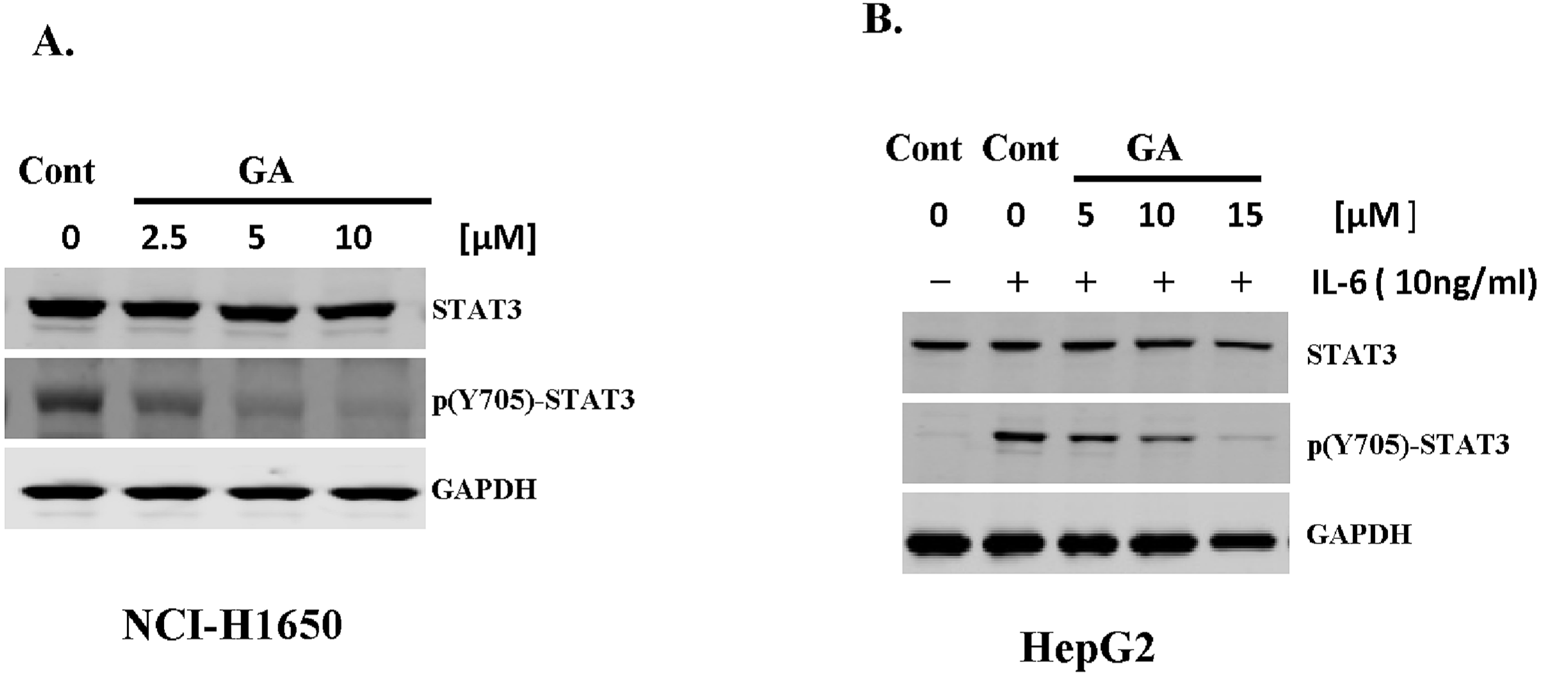
A.
Cont
GA
0
2.5
5
10
[μM]
STAT3
p(Y705)-STAT3
GAPDH
NCI-H1650
B.
Cont
Cont
GA
0
0
5
10
15
[μM]
−
+
+
+
+
IL-6 (10ng/ml)
STAT3
p(Y705)-STAT3
GAPDH
HepG2
